# Eplerenone in patients with myocardial infarction and “mid‐range” ejection fraction: An analysis from the EPHESUS trial

**DOI:** 10.1002/clc.23261

**Published:** 2019-09-03

**Authors:** João Pedro Ferreira, Xavier Rossello, Bertram Pitt, Patrick Rossignol, Faiez Zannad

**Affiliations:** ^1^ Université de Lorraine, Centre d'Investigation Clinique‐ Plurithématique Inserm CIC‐P 1433 Nancy France; ^2^ Inserm U1116, CHRU Nancy Brabois, F‐CRIN INI‐CRCT (Cardiovascular and Renal Clinical Trialists) Nancy France; ^3^ Centro Nacional de Investigaciones Cardiovasculares (CNIC) Madrid Spain; ^4^ Centro de Investigación Biomédica en Red en Enfermedades Cardiovasculares (CIBERCV) Madrid Spain; ^5^ Department of Medicine University of Michigan School of Medicine Ann Arbor Michigan

**Keywords:** ejection fraction, eplerenone, mineralocorticoid receptor antagonists, myocardial infarction, treatment effect

## Abstract

**Background:**

Trials using mineralocorticoid receptor antagonists (MRAs) in myocardial infraction (MI) without heart failure (HF) or systolic impairment have been underpowered to assess morbidity‐mortality benefit. In EPHESUS 6632 patients were included, of whom 11% had an ejection fraction (EF) of 40% and HF or diabetes. We aim to assess the potential benefit of MRAs in MI with EF of 40%.

**Methods:**

Cox models with interaction term for EF. The primary outcome was a composite of cardiovascular death or hospitalization for cardiovascular reasons.

**Hypothesis:**

Patients with an EF of 40% benefit similarly from MRA therapy to those with an EF <40%.

**Results:**

In EPHESUS, 753 patients had an EF = 40% and 5864 an EF < 40%. Patients with an EF = 40% were younger (63 vs 64 years), had lower heart rate (73 vs 75 bpm), less atrial fibrillation (10% vs 14%), previous MI (21% vs 28%), HF hospitalization (5% vs 8%), and had more often reperfusion therapy and/or revascularization (55% vs 44%). The mean EF was 40.0 ± 0.3% in those with EF = 40% vs 32.2 ± 5.9% in those with EF < 40%. The primary outcome occurred in 13.3% (10 events per 100 py) of the patients with EF = 40% vs 22.9% (19 events per 100 py) in those with EF < 40%; adjusted HR for EF = 40% vs <40% = 0.65 (0.53‐0.81). Eplerenone reduced the event‐rate homogenously regardless of EF (interaction_*p*_ EF = 40% vs EF < 40% = 0.21). Similar findings were observed for cardiovascular and all‐cause death.

**Conclusion:**

Eplerenone reduces hospitalizations and mortality in patients with MI and EF = 40% similarly to patients with EF < 40%. These findings suggest that MI patients with EF in the “mid‐range zone” may also benefit from MRA therapy which might help clinicians in their treatment decisions.

## 1 INTRODUCTION

Mineralocorticoid receptor antagonists (MRAs) have clear “IA” guideline indication for patients with a myocardial infraction (MI) and a left ventricular ejection fraction (EF) inferior to 40% if accompanied by signs and symptoms of heart failure (HF) or diabetes.^1^, ^2^ Despite the guideline recommendations, MRAs are prescribed in a small proportion of these patients.^3^, ^4^

In patients with an EF of 40% or greater, the potential benefit of MRAs is unknown. A large proportion of patients with a recent MI have a EF of 40% or greater regardless of having (or not) signs and symptoms of HF. In the HF field, patients with an EF situated between 40% and 50% have been called “mid‐range” EF patients,^1^ and there is some evidence suggesting that they might benefit from MRA therapy.^5^, ^6^ However, in MI this specific population has not been studied in dedicated trials using MRAs, and trials including patients without HF at presentation have been underpowered to ascertain morbidity‐mortality benefit.^7^, ^8^

In the Eplerenone, a Selective Aldosterone Blocker, in Patients with Left Ventricular Dysfunction after Myocardial Infarction (EPHESUS) trial,^9^ eplerenone reduced the relative rate of all‐cause death by 15% and the composite of death from cardiovascular causes or hospitalization for cardiovascular events by 13%. In EPHESUS, patients could be enrolled within 3 to 14 days after a MI, if they had an EF ≤ 40% and HF or diabetes. In this large trial, it is thus possible that many of these patients had an EF of 40% or greater due to a “digit preference” for EF values in multiples of 5%, as it has been described in previous reports that patients with EF between 35% and 45% are attributed to have an EF of 40%.^10^, ^11^

We performed an analysis of the EPHESUS trial to assess the characteristics, event‐rates and the effect of eplerenone in patients with EF = 40%, compared with those with EF < 40%.

## 2 METHODS

### 2.1 Study design, setting, and participants

EPHESUS included 6632 patients with an acute MI (3‐14 days after the MI), and a LVEF ≤ 40% plus HF or diabetes to receive either eplerenone (25 mg per day initially, titrated to a maximum of 50 mg per day) or placebo. Among all enrolled patients, 743 (11.2%) and an EF = 40%, and only 10 had an EF > 40%. Therefore, we refer to these patients as having an EF of 40%.

The primary end points were (a) a composite of death from cardiovascular causes or hospitalization for cardiovascular causes (including HF, acute MI, stroke, or ventricular arrhythmia), and (b) death from any cause. The median (percentile 25‐75) follow‐up time was 1.3 (1.0‐1.7) years.^9^

EPHESUS was conducted in accordance with the Declaration of Helsinki and approved by the site ethics committees. All participants gave written informed consent to participate in the study.

### 2.2 Study outcomes

In the present analysis, the primary outcome is a composite of cardiovascular death or cardiovascular hospitalization. Cardiovascular death alone, all‐cause death, and HF hospitalization were also assessed. Hyperkalemia (defined as a potassium concentration above 5.5 mmol/l at any time throughout the follow‐up) and worsening renal function (WRF, defined as a decline superior to 30% in the estimated glomerular filtration rate at any time throughout the follow‐up) were assessed as major safety outcomes.

The outcomes were centrally adjudicated by endpoint committees and defined by the conventional criteria.^9^

### 2.3 Statistical analysis

Baseline clinical characteristics of patients were summarized by EF groups (<40% vs 40%) using means and SD for continuous variables, frequencies and percentages for categorical variables, and hazard ratios (HRs), incidence rates, and incidence‐rate differences with their 95% confidence interval (95%CI) for treatment effect estimates.

Time‐to‐first‐event curves were obtained using the Kaplan‐Meier method and compared using the log‐rank test. Univariate Cox proportional hazards' modeling was used to explore the association between EF and/or the intervention and the study outcomes, and a Cox model with interaction term on EF was performed to assess the potential heterogeneity of the treatment effect by EF. We also performed adjusted models for reducing the potential confounding of the associations between EF and outcomes. The covariates for this multivariate model were chosen according to their clinical relevance or historical association with the outcome in the previous studies^12^, ^13^, ^14^ and included age, sex, body mass index, heart rate, estimated glomerular filtration rate, sodium, potassium, previous MI, peripheral vascular disease, previous HF hospitalization, atrial fibrillation, diabetes, hypertension, chronic obstructive pulmonary disease, angiotensin‐converting‐enzyme‐inhibitors/angiotensin‐receptor blockers, beta‐blockers, Q‐wave MI, Killip class, reperfusion therapy, and study drug (eplerenone or placebo). Proportional hazards assumptions were tested based on Schoenfeld residuals with time interaction.

All the statistical analyses were performed using the STATA/SE software, version 15.1 (Stata Corp, College Station, Texas).

## 3 RESULTS

### 3.1 Patients' characteristics

In the present analysis, 753 patients had an EF = 40% and 5864 an EF < 40%. Patients with EF = 40% were slightly younger (63 vs 64 years), had lower heart rate (73 vs 75 bpm), lower proportion of atrial fibrillation (10 vs 14%), previous MI (21 vs 28%), HF hospitalization (5 vs 8%), and had more often reperfusion therapy and/or revascularization (55 vs 44%) (Table 1). The mean EF was 40.0 ± 0.3% in those with EF = 40% vs 32.2 ± 5.9% in those with EF < 40%. Only 10 patients (0.1%) had an EF > 40% (Supporting Information Figure 1).

**Table 1:** Baseline clinical features by EF categories

Characteristics	EF < 40%	EF = 40%	*p* value
*N*	5864	753	
Age, years	64.0 ± 11.5	63.1 ± 11.5	.031
Male sex	4164 (71.0%)	536 (71.2%)	.92
White race	5287 (90.2%)	682 (90.6%)	.42
Smoking	1800 (30.7%)	242 (32.2%)	.69
SBP, mmHg	118.9 ± 16.5	120.3 ± 16.5	.028
DBP, mmHg	72.0 ± 10.7	72.8 ± 10.6	.067
Heart rate, bpm	74.9 ± 11.8	73.4 ± 10.7	<.001
BMI, kg/m^2^	27.4 ± 4.5	27.7 ± 4.6	.087
Diabetes	1895 (32.3%)	239 (31.7%)	.75
Hypertension	3535 (60.3%)	460 (61.1%)	.67
Atrial fibrillation	792 (13.5%)	78 (10.4%)	.016
MI before index AMI	1637 (27.9%)	155 (20.6%)	<.001
Angina	2453 (41.8%)	276 (36.7%)	.007
Peripheral vascular disease	730 (12.4%)	89 (11.8%)	.62
HFH before index AMI	464 (7.9%)	40 (5.3%)	.011
COPD	553 (9.4%)	69 (9.2%)	.81
eGFR, ml/min/1.73 m^2^	68.3 ± 20.9	69.3 ± 20.7	.21
Hemoglobin, g/dl	13.3 ± 1.7	13.3 ± 1.7	.99
Potassium, mmol/l	4.3 ± 0.5	4.3 ± 0.4	.30
Sodium, mmol/l	139.4 ± 4.2	140.0 ± 5.2	<.001
ACEI/ARB	5112 (87.2%)	627 (83.3%)	.003
Beta‐blocker	4359 (74.3%)	591 (78.5%)	.014
Index Q‐wave AMI	4153 (72.2%)	549 (73.8%)	.37
Killip class I	846 (14.5%)	163 (21.7%)	<.001
II	3800 (65.2%)	469 (62.5%)	
III	995 (17.1%)	96 (12.8%)	
IV	184 (3.2%)	23 (3.1%)	
Reperfusion or revascularization	2585 (44.1%)	416 (55.2%)	<.001
EF, %	32.2 ± 5.9	40.0 ± 0.3	<.001
Time from AMI to rand., days	7.3 ± 3.0	7.0 ± 3.1	.032
Eplerenone	2916 (49.7%)	397 (52.7%)	.12
Duration of hosp. for index AMI, days	15.4 ± 10.2	13.9 ± 7.7	<.001

Abbreviations: ACE/ARB, angiotensin converting enzyme inhibitor or angiotensin receptor blocker; AMI, acute myocardial infarction; COPD, chronic obstructive pulmonary disease; CV, cardiovascular; DBP, diastolic blood pressure; EF, left ventricular ejection fraction; eGFR, estimated glomerular filtration rate calculated by the CKD‐EPI formula; HF, heart failure; MI, myocardial infarction; SBP, systolic blood pressure.

### 3.2 Events by left ventricular EF categories

Patients with an EF = 40% had lower event rates compared to patients with an EF < 40%. The primary outcome occurred in 13.3% of the patients with an EF = 40% vs 22.9% in those with an EF < 40%, with corresponding event rates per 100 person‐years of 10.3 (8.5‐12.5) vs 19.2 (18.2‐20.2). The adjusted HR (95%CI) for EF = 40% when compared to patients with EF < 40% was 0.65 (0.53‐0.81).

All cause‐death occurred in 9.0% of the patients with an EF = 40% vs 16.4% in those with an EF < 40%, with corresponding event rates per 100 person‐years of 6.6 (5.2‐8.4) vs 12.6 (11.8‐13.4). The adjusted HR (95%CI) for EF = 40% compared with EF < 40% as reference was 0.65 (0.50‐0.83). Consistent findings were observed for cardiovascular death and HF hospitalization (Table 2).

**Table 2:** Events by EF categories

Outcome	*N* (%) events	Event rates	Crude HR (95%CI)	*p* value	Adj. HR (95%CI)^a^	*p* value
*Primary outcome*						
EF < 40%	1344 (22.9)	19.2 (18.2–20.2)	Ref.	<.001	Ref.	<.001
EF = 40%	100 (13.3)	10.3 (8.5–12.5)	0.55 (0.44‐0.67)		0.65 (0.53‐0.81)	
*CV death*						
EF < 40%	833 (14.2)	10.9 (10.2‐11.7)	Ref.	<.001	Ref.	<.001
EF = 40%	52 (6.9)	5.0 (3.8‐6.6)	0.47 (0.35‐0.62)		0.58 (0.43‐0.77)	
*All‐cause death*						
EF < 40%	959 (16.4)	12.6 (11.8–13.4)	Ref.	<.001	Ref.	.001
EF = 40%	68 (9.0)	6.6 (5.2–8.4)	0.53 (0.41‐0.68)		0.65 (0.50–0.83)	
*HF hospitalization*						
EF < 40%	787 (13.4)	11.2 (10.5‐12.0)	Ref.	<.001	Ref.	.008
EF = 40%	64 (8.5)	6.6 (5.2–8.4)	0.60 (0.46‐0.77)		0.70 (0.53‐0.91)	

*Note*: The primary outcome is a composite of cardiovascular death or cardiovascular hospitalization. Median follow‐up time to death or censor = 1.3 (1.0‐1.7) years. Event rates per 100 person‐years.

Abbreviations: CV, cardiovascular; EF, left ventricular ejection fraction; HF, heart failure.

a Model adjusted on age, sex, body mass index, heart rate, estimated glomerular filtration rate, sodium, potassium, previous myocardial infarction, peripheral vascular disease, previous heart failure hospitalization, atrial fibrillation, diabetes, hypertension, chronic obstructive pulmonary disease, angiotensin‐converting‐enzyme‐inhibitors/angiotensin‐receptor blockers, beta‐blockers, Q‐wave myocardial infarction, Killip class, reperfusion therapy, and study drug (eplerenone or placebo).

### 3.3 Treatment effect

**Figure 1:**
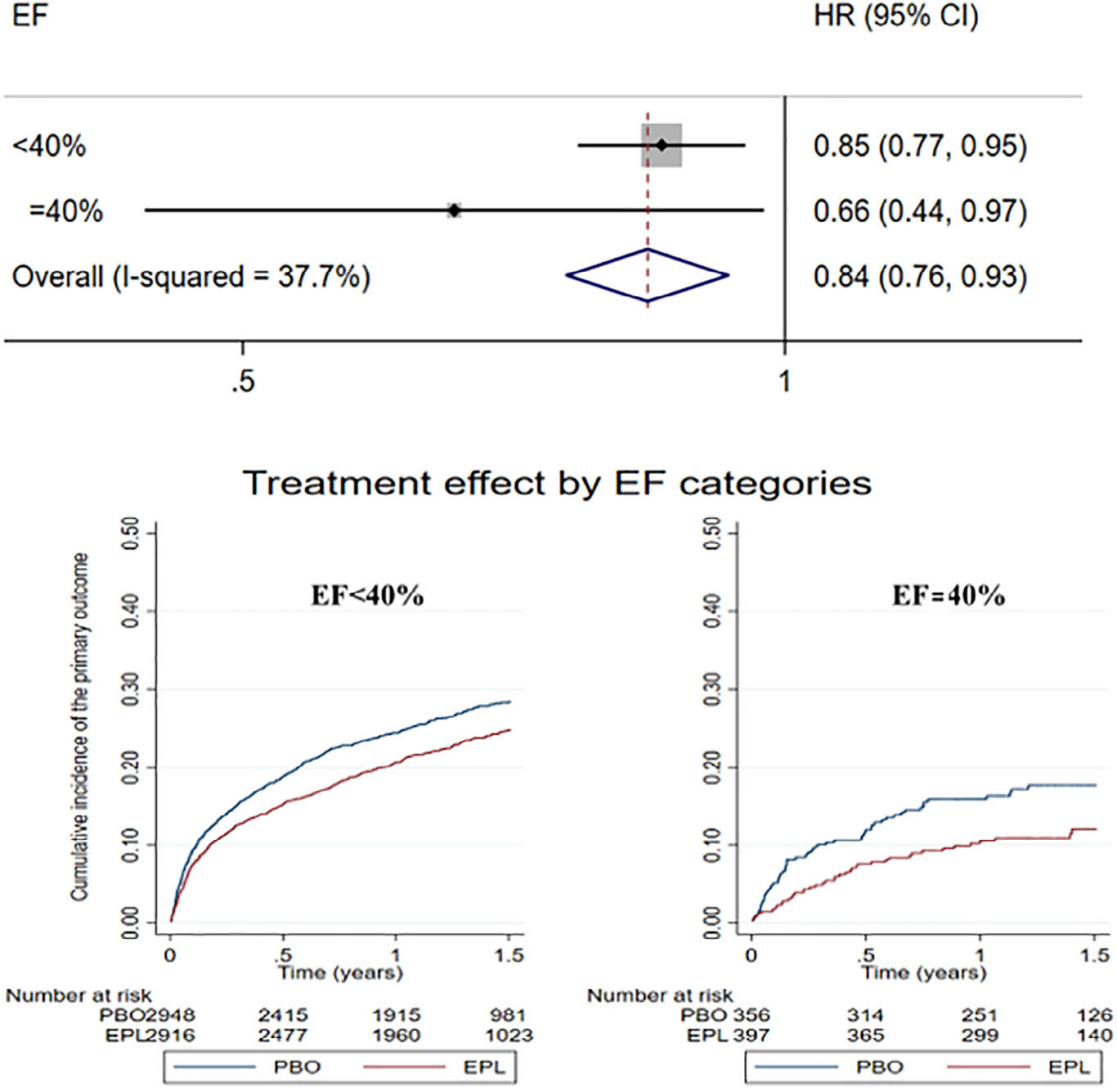
Forest plot with hazard ratios and 95% confidence intervals and Kaplan‐Meier curves for the outcome of cardiovascular death or cardiovascular hospitalization by EF categories. P for interaction by EF category (<40% vs 40%) = 0.21. The primary outcome is a composite of cardiovascular death or cardiovascular hospitalization. Median follow‐up time = 1.3 (1.0‐1.7) years. EF, left ventricular ejection fraction; EPL, eplerenone; PBO, placebo

Eplerenone (compared with placebo) reduced the event rates with similar magnitude regardless of the EF, with relative reductions of the primary outcome ranging from 2% to 56% in patients with EF = 40% and 5% to 23% in patients with EF < 40%; *p* for interaction = 0.21. The effect of treatment was more imprecise in patients with EF = 40% due to a smaller sample size/loss of statistical power. All the studied outcomes pointed toward a benefit of eplerenone regardless of the EF (Table 3 and Figure 1).

**Table 3:** Treatment effect by EF categories

Outcome	Event rates PBO	Event rates EPL	HR (95%CI)	Interaction_*p*_
*Primary outcome*				
EF < 40%	20.8 (19.3‐22.4)	17.6 (16.2‐19.0)	0.85 (0.77‐0.95)	0.21
EF = 40%	12.7 (9.8‐16.4)	8.3 (6.1‐11.1)	0.66 (0.44‐0.98)	
*CV death*				
EF < 40%	11.9 (10.9‐13.1)	10.0 (9.0‐11.0)	0.84 (0.73‐0.96)	0.39
EF = 40%	6.2 (4.3‐8.8)	4.0 (2.7‐6.1)	0.66 (0.38‐1.13)	
*All‐cause death*				
EF < 40%	13.6 (12.5‐14.9)	11.6 (10.6‐12.7)	0.85 (0.75‐0.97)	0.60
EF = 40%	7.6 (5.5‐10.5)	5.7 (4.0‐8.1)	0.75 (0.46‐1.21)	
*HF hospitalization*				
EF < 40%	12.1 (11.0‐13.3)	10.4 (9.4‐11.5)	0.87 (0.75‐1.00)	0.23
EF = 40%	8.2 (6.0‐11.4)	5.2 (3–6‐7.6)	0.63 (0.39‐1.04)	

*Note*: The primary outcome is a composite of cardiovascular death or cardiovascular hospitalization. Median follow‐up time to death or censor = 1.3 (1.0‐1.7) years. Event rates per 100 person‐years.

Abbreviations: CV, cardiovascular; EPL, eplerenone; HF, heart failure; LVEF, left ventricular ejection fraction; PBO, placebo.

**Figure 2:**
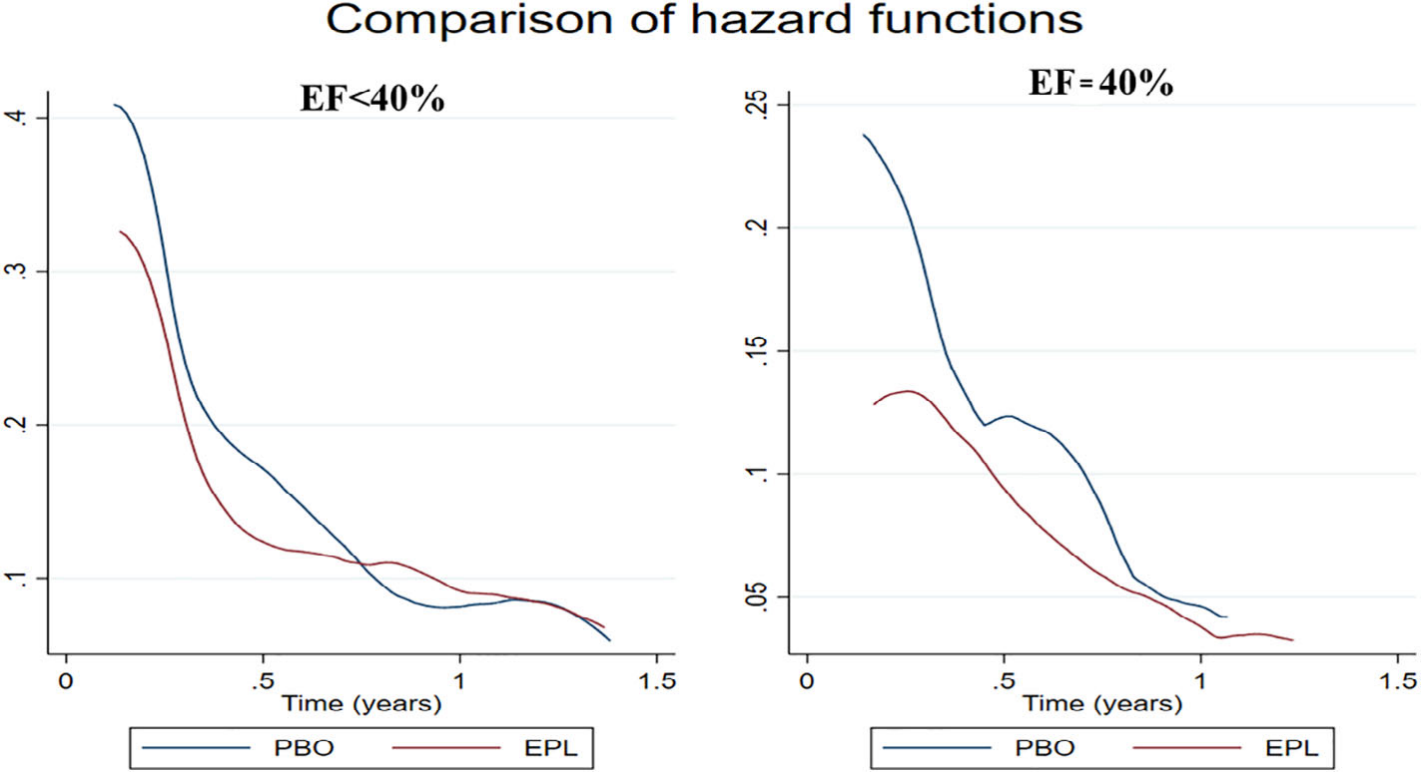
Smoothed hazard estimates of the treatment effect over time on the study primary outcome. The treatment effect is more marked in the early follow‐up period, regardless of the EF. The primary outcome is a composite of cardiovascular death or cardiovascular hospitalization. Median follow‐up time = 1.3 (1.0‐1.7) years. EF, left ventricular ejection fraction; EPL, eplerenone; PBO, placebo

The effect of treatment was of higher absolute magnitude in the early follow‐up period (first 6 months), regardless of EF (Figure 2 and Supporting Information Table 1). Proportional hazards assumptions can be fairly assumed (proportional‐hazards test based on Schoenfeld residuals *p*‐value in patients with an EF = 40% = 0.24 and in EF < 40% = 0.13).

### 3.4 Side effects

Hyperkalemia (K^+^ > 5.5 mmol/l) and WRF (defined as a decline in eGFR > 30%) at any time throughout the follow‐up, occurred similarly between the eplerenone and placebo groups in patients with an EF = 40% (hyperkalemia: 14.8% in eplerenone vs 13.1% in placebo; *p*‐value = 0.53, and WRF: 17.7% in eplerenone vs 15.8% in placebo; *p*‐value = 0.49). In patients with EF < 40% hyperkalemia and WRF occurred more frequently in patients taking eplerenone (hyperkalemia: 15.9% in eplerenone vs 11.4% in placebo; *p*‐value < .001, and WRF: 23.0% in eplerenone vs 19.5% in placebo; *p*‐value = .001); but without statistically significant differences (interaction) by EF (interaction_*p*_ = 0.27 for hyperkalemia and = 0.72 for WRF).

## 4 DISCUSSION

In EPHESUS, treatment with eplerenone (vs placebo) significantly reduced the composite of time‐to‐first cardiovascular death or cardiovascular hospitalization, cardiovascular and all‐cause death, and HF hospitalization, regardless of the EF (40% vs <40%). These findings support the use of eplerenone also in MI patients with EF of 40%.

In HF, patients enrolled in the Spironolactone for Heart Failure with Preserved Ejection Fraction (TOPCAT) trial, who had a “mid‐range” EF, might have benefited more from spironolactone therapy.^5^, ^6^ Notwithstanding, these HF patients were substantially different from the MI patients enrolled in EPHESUS. To the best of our knowledge, no study has demonstrated the efficacy of MRAs on morbidity‐mortality end‐points in patients with a EF ≥ 40% in MI patients. The role of MRAs in MI without systolic dysfunction or HF has been evaluated in two randomized controlled trials: the Early Aldosterone Blockade in Acute Myocardial Infarction (ALBATROSS)^8^ and the Early Eplerenone Treatment in patients with acute ST‐elevation Myocardial Infarction without Heart Failure (REMINDER)^7^ trials. These trials demonstrated the safety of MRA therapy in this setting but did not reduce morbidity nor mortality, likely because the trials were underpowered to assess the treatment effect in terms of major cardiovascular events (such as death or hospitalizations). Consequently, until larger and adequately powered trials are conducted, MRA therapy cannot be routinely advised in post‐MI patients without systolic dysfunction and/or HF. An individual patient‐data meta‐analysis of the ALBATROSS and REMINDER trials pointed toward a potential mortality benefit of MRA use in a MI population without HF, but even pooling these trials the event rates was too low to draw any solid conclusions.^15^ Notwithstanding, based on the findings depicted herein, MRAs could be considered in patients with a MI and an EF around 40%. The event rate reduction is marked in the first few months of therapy initiation (and may be maintained thereafter), this is of particular relevance because MRAs may reduce the risk of sudden death in this “high‐risk” post‐MI period were defibrillators are not indicated.^14^, ^16^ The ongoing Colchicine and Spironolactone in Patients With STEMI/SYNERGY Stent Registry (CLEAR‐SYNERGY) trial (http://clinicaltrials.gov Identifier: NCT03048825) may help in assessing the potential effect of MRAs in patients with MI and an EF ≥ 40%.

In our study, and as previously documented,^9^ there was an increased incidence of hyperkalemia and WRF among patients receiving eplerenone (additionally, our study suggests that these side effects might have been experienced mainly by patients with an EF < 40%). This finding underscores the need to measure serum potassium and creatinine levels serially and to adjust the dose of eplerenone accordingly. In EPHESUS, the protocol mandated “if at any time during the study the serum potassium is >5.5 mEq/l but <6.0 mEq/l, the dose of study drug will be reduced to the next lower dose level or temporarily withheld if the patient is receiving 25 mg of eplerenone every other day.^9^ In a renal function stratified analysis from the EMPHASIS‐HF trial, we found that a dose of 25 mg/day of eplerenone in patients with eGFR <50 ml/min is as effective as 50 mg/day in patients with eGFR ≥ 50 ml/min; these are the doses that should be used in clinical practice (ie, personalized approach) because, by adapting the dose according to the renal function, one may avoid side‐effects and drug‐discontinuation.^17^

## 5 LIMITATIONS

This is a post hoc nonprespecified analysis of the EPHESUS trial. In consequence, these results are prone to bias inherent to secondary studies and should be regarded as exploratory and hypothesis generating. Moreover, EPHESUS was not designed with sufficient power to draw statistical conclusions about subgroups (baseline characteristics were well balanced between MRA and placebo groups, but they were not between EF < 40 and EF = 40%). We have addressed this issue by adjusting for potential confounders. Generally, the absence of significant “interactions” in all the studied outcomes, points toward a consistent eplerenone effect regardless of the EF subgroup.

## 6 CONCLUSIONS

Eplerenone reduces hospitalizations and mortality in patients with EF of 40% similarly to patients with EF < 40%. These findings suggest that MI patients with EF in the “mid‐range zone” may also benefit from MRA therapy which might help clinicians in their treatment decisions.

## CONFLICT OF INTEREST

The authors declare no potential conflict of interests.

## Supporting information


**Data S1** Supporting Information Table 1 and Figure 1.
