# Supplementary material for: Eplerenone in patients with myocardial infarction and “mid‐range” ejection fraction: An analysis from the EPHESUS trial
Source: Clin Cardiol. 2019 Sep 3;42(11):1106–12. doi: 10.1002/clc.23261 (PMC6837025; doi:10.1002/clc.23261)
Supplement: Supplementary file 1 — Data S1 Supporting Information Table 1 and Figure 1. [file CLC-42-1106-s001.docx]

**Supplemental Material**

Supplemental Table 1. Treatment effect on the primary outcome by EF categories over time

| **Outcome** | **Event rates PBO** | **Event rates EPL** | **IRD (95%CI)** | **P-value** |
| --- | --- | --- | --- | --- |
| **0-6 months** | | | | |
| EF<40% | 39.1 (35.8-42.6) | 31.5 (28.6-34.7) | -7.6 (-12.4 to -3.1) | 0.001 |
| EF=40% | 24.5 (17.9-33.3) | 15.3 (10.7-22.1) | -9.2 (-18.1 to 0.8) | 0.072 |
| **6-12 months** | | | | |
| EF<40% | 11.5 (9.7-13.7) | 10.9 (0.2-13.0) | -0.6 (-3.3 to 2.1) | 0.69 |
| EF=40% | 8.2 (4.7-14.4) | 5.8 (3.1-10.8) | -2.5 (-8.9 to 2.9) | 0.32 |
| **>12 months** | | | | |
| EF<40% | 8.3 (6.7-10.2) | 8.0 (6.5-9.9) | -0.3 (-2.7 to 2.1) | 0.83 |
| EF=40% | 3.6 (1.5-8.6) | 2.5 (0.9-6.7) | -1.1 (-5.0 to 2.9) | 0.61 |

Legend: LVEF, left ventricular ejection fraction; CV, cardiovascular; HF, heart failure; PBO, placebo; EPL, eplerenone; IRD, incidence rate difference.

The primary outcome is a composite of cardiovascular death or cardiovascular hospitalization.

Median follow-up time = 1.3 (1.0-1.7) years.

Event rates per 100 person-years.

Supplemental Figure 1. EF density plots

1. EF≥40%


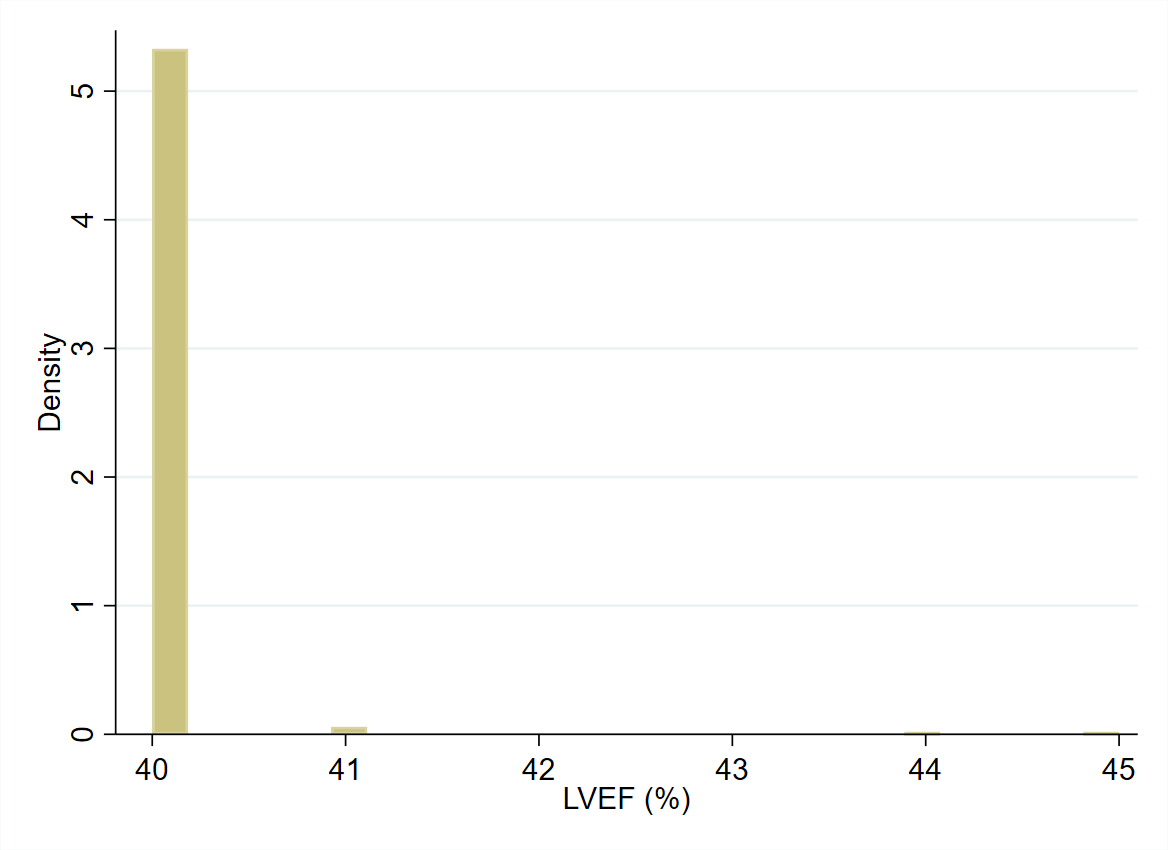


1. EF<40%


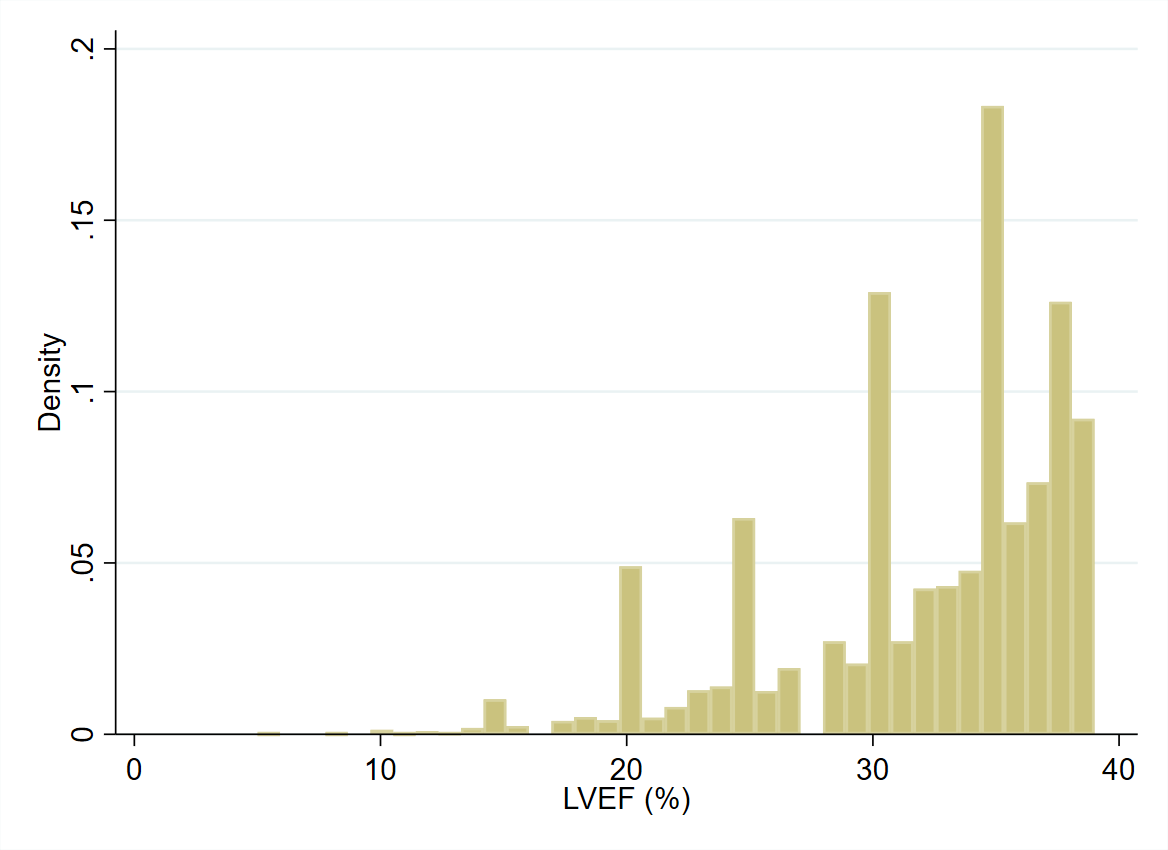


Most patients with EF≥40% had an EF equal to 40% (n =743; 99%).

Legend: EF, left ventricular ejection fraction.
